# The effects of heated humidifier in continuous positive airway pressure titration

**DOI:** 10.1007/s11325-012-0661-y

**Published:** 2012-02-04

**Authors:** Chung-Chieh Yu, Cheng-Ming Luo, Yu-Chih Liu, Huang-Pin Wu

**Affiliations:** 1 Division of Pulmonary, Critical Care, and Sleep Medicine, Chang Gung Memorial Hospital, Keelung, Taiwan Republic of China; 2 Chang Gung University College of Medicine, Taoyuan, Taiwan Republic of China; 3 Department of Respiratory Therapy, Chang Gung Memorial Hospital, Keelung, Taiwan Republic of China; 4 Department of Otolaryngology-Head and Neck Surgery, Chang Gung Memorial Hospital, Keelung, Taiwan Republic of China; 5 Division of Pulmonary Medicine, Department of Internal Medicine, Chang Gung Memorial Hospital, Keelung #222, Maijin Rd., Anle Chiu, Keelung, Taiwan 204 Republic of China

**Keywords:** Continuous positive airway pressure (CPAP) titration, Humidifier, Obstructive sleep apnea–hypopnea syndrome

## Abstract

**Background:**

Previous studies have shown that routine heated humidifier (HH) do not provide any benefit during continuous positive airway pressure (CPAP) titration if there are no significant naso-pharyngeal symptoms. In clinical practice, nasal diseases and upper airway symptoms are very common. This study investigates the effects of HH during CPAP titration in subjects with or without naso-pharyngeal symptoms.

**Methods:**

Fifty-two patients who received polysomnography with CPAP titration were randomly assigned to HH and non-HH groups. Their nasal cavity, pharynx, and naso-pharynx were evaluated before CPAP titration, and a questionnaire on subjective sensation, including naso-pharyngeal symptoms, willingness to further use CPAP, and sleep improvement, was used. Objective (e.g., leak, apnea–hypopnea index (AHI) reduction, and optimal CPAP pressure level) and subjective data were analyzed between the two groups.

**Results:**

In subjective sensation, the HH group did not have any benefit in further willingness to use CPAP and in sleep improvement, but had improved naso-pharyngeal symptoms (*p* = 0.043). There were no significant differences in leak, AHI reduction, and optimal CPAP pressure, even in patients with significant naso-pharyngeal symptoms.

**Conclusion:**

Routine use of HH is not necessary during CPAP titration regardless of naso-pharyngeal symptoms.

## Introduction

Obstructive sleep apnea–hypopnea syndrome (OSAHS) is a common medical condition, with an incidence of 4–7% in the adult general population [1]. Nasal continuous positive airway pressure (CPAP) is the mainstay of treatment, and its effectiveness is well documented [2]. Practical guideline suggests undergoing attended CPAP titration before home CPAP therapy [3]. However, CPAP can be associated with troublesome side effects, including nasal congestion, sore throat, and dry nose and throat [4–6]. Some reports reveal that the use of heated humidifier (HH) can alleviate upper airway symptoms and improve CPAP adherence [7, 8].

The degree of sleep improvement during CPAP titration may be a crucial factor in determining the subsequent use of this treatment modality [9–11]. Duong’s study has shown that using HH during CPAP titration offers no additional benefit both in acceptance and naso-pharyngeal symptoms. However, this study excludes subjects with significant naso-pharyngeal symptoms [12]. In contrast, several studies reveal that using HH from weeks to months can reduce naso-pharyngeal symptoms [13–15]. In clinical practice, OSAHS patients have high prevalence of nasal diseases and upper airway symptoms. The role of HH during CPAP titration is not clear in enrolled patients with naso-pharyngeal symptoms, and there is still no consensus about the use of HH during CPAP titration. Thus, this study was conducted to investigate the HH effects during CPAP titration.

## Methods

### Subjects

Patients newly diagnosed with OSAHS were enrolled. OSAHS was defined by initial diagnostic polysomnography with an apnea–hypopnea index (AHI) >15/h. Patients with co-existing congestive heart failure, central sleep apnea, obesity hypoventilation, and chronic obstructive airway disease were excluded. Patients who refused CPAP titration or who could not complete CPAP titration were also excluded. The institutional review board of Chang Gung Memorial Hospital approved the study, and all subjects signed inform consent.

### Study design

This was a randomized control study. Before CPAP titration, all subjects were referred to the otorhinolaryngology clinic for evaluation of the nasal cavity, pharynx, and naso-pharynx. All patients underwent manual CPAP titration to determine the optimal CPAP level. They were then randomly assigned to two groups. One group used HH (H4i, ResMed, Sydney, Australia) during CPAP titration, and the other group did not use HH but underwent titration with the same CPAP machine and humidifier devices. The technician did not provide any information about the humidifier to the study subjects.

The humidification level was applied according to the manufacturer’s manual (level 2–3) and adjusted accordingly based on temperature and water condensation. Subjects completed questions regarding naso-pharyngeal symptoms before and after CPAP titration and documented their willingness to use CPAP and feelings of sleep improvement after titration.

### Definition of nasal disease

All of the subjects were evaluated by an otorhinolaryngologist. Patients with positive nasal symptoms were evaluated further for blood IgE and allergen test. The diagnostic criteria of nasal diseases were as follows:

1. Rhino-sinusitis. The symptoms for clinical evaluation included major and minor criteria. For acute rhino-sinusitis, the major criteria included purulent discharge, headache, facial pain or pressure, nasal congestion, decreased smelling sensation, and fever while the minor criteria included halitosis, fever, weakness, dental pain, ear fullness and pain, and cough. For chronic rhino-sinusitis, the bases were two or more of major criteria or one major and two minor criteria in a 12-week history [16].
2. Allergic rhinitis. The diagnosis was confirmed with a positive history and specific allergens or the symptoms from IgE-mediated inflammation [17].
3. Non-allergic rhinitis. It was defined by chronic nasal symptoms without concomitant allergic disease as determined by allergen-specific antibody tests [18].
4. Nasal septum deviation. It was defined as a deviation of more than 4 mm from the midline [19].

### Polysomnography and CPAP titration

Standard overnight polysomnography included recorded electroencephalography, bilateral electrooculograms, submental electromyogram, electrocardiography, nasal and oro-nasal airflow (nasal pressure and thermistor), oximetry, chest and abdominal movements (inductance plethysmography), body position, sound intensity, and bilateral tibial electromyogram. All signals were collected and digitized on a computerized polysomnography system (N7000 Embla, Broomfield, USA). Sleep stages were scored in 30-s epochs, using the Rechtschaffen and Kales sleep scoring criteria [20].

Apnea was defined as the absence of airflow for 10 s. Obstructive apnea was defined as the absence of airflow in the presence of rib cage and/or abdominal excursions, while central apnea was defined as the absence of airflow and rib cage and abdominal excursions. Hypopnea was considered for any visible reduction in airflow >50% lasting at least 10 s and associated with either a 3% decrease in arterial oxyhemoglobin saturation or an appearance of electroencephalogram arousal [21]. The AHI was defined as the average number of apneas and hypopneas per hour of sleep.

All patients underwent nasal CPAP titration study in the sleep laboratory in a separate night. In the CPAP titration study, manual CPAP titration was done to determine the optimal CPAP level [22]. A CPAP interface was individually fitted from a wide range of interfaces to maximize comfort and minimize leak. All participants used an AutoSet Spirit S8 (ResMed, Sydney, Australia) throughout the study. A technician who supervised the study also prepared the patients and corrected the mask position and fitting initially. The lowest CPAP pressure (4 cm H_2_O) was applied to the patients initially and increased when the patients went to sleep. The optimal CPAP pressure was determined when the pressure could eliminate apnea, hypopnea, desaturation, and snoring in a supine position and considered “better” in rapid-eye movement sleep. Leak values, including median leak, 95th percentile leak (leak level covering 95% of the study period), and maximum leak, were read from the AutoSet Spirit S8 by the software of AutoScan 5.7 (ResMed, Sydney, Australia).

### Questions for naso-pharyngeal symptoms and subjective feeling

**Fig. 1:**
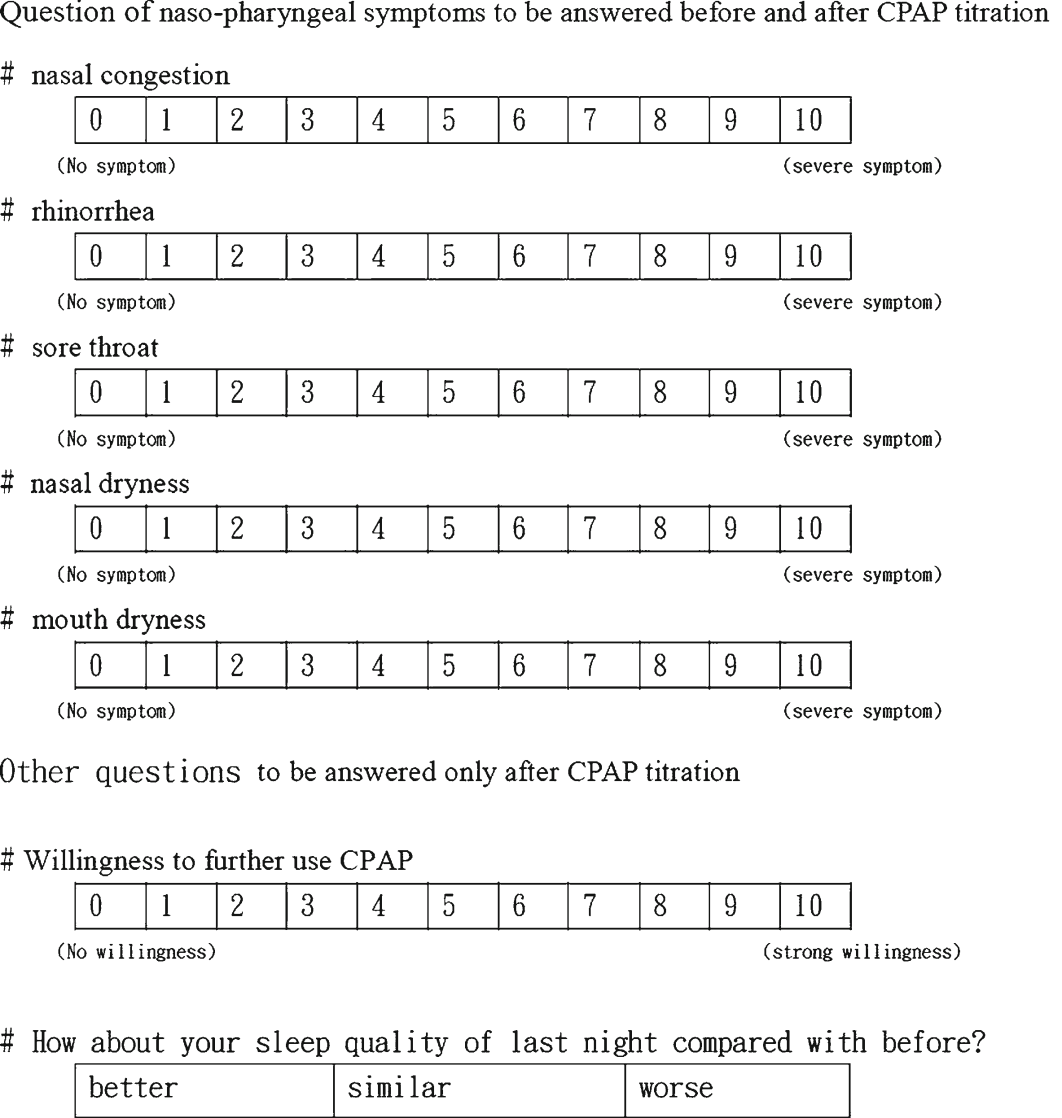
Questions for naso-pharyngeal symptoms and subjective feeling

Patients completed the questions of naso-pharyngeal symptoms score before and after CPAP titration. Questions on nasal–pharyngeal symptoms involved five items (nasal congestion, rhinorrhea, sore throat, and nasal and mouth dryness) presented via visual analog scale rated on ten-point scale. There were also questions about willingness to use CPAP and on sleep improvement sensation after completing CPAP titration (Fig. 1). Total upper airway symptom (TUAS) is defined the sum of total score of nasal congestion, rhinorrhea, sore throat, and nasal and mouth dryness.

The study also compared optimal CPAP pressure, leak values, degree of AHI, willingness to further use CPAP, sleep improvement, and naso-pharyngeal symptoms between the two groups.

### Statistical analysis

Statistical evaluations were performed using the SPSS Software (SPSS Inc., Chicago, IL). Data were presented as mean ± standard deviation (SD). Comparison between leak, AHI reduction, optimal CPAP pressure, naso-pharyngeal symptoms, willingness to use CPAP, and sleep improvement sensation were done using the Mann–Whitney *U* test. A *p* < 0.05 was considered statistically significant.

## Results

Of the 57 patients enrolled, five were excluded (two with extremely high AHI, one with incomplete CPAP titration, and two who did not complete the questionnaires). The 52 subjects were randomly divided to the HH and non-HH groups. Their basic characteristics, including important baseline data such as AHI, body mass index, age, Epworth sleepiness score, and gender, were similar (Table 1). The prevalence of nasal diseases was high. Chronic mucosal diseases, including rhino-sinusitis (*n* = 8), allergic rhinitis (*n* = 18), and non-allergic rhinitis (*n* = 14), were as high as 77% (*n* = 40). Anatomic diseases like nasal septum deviation also had a high incidence of 48% (*n* = 25). Twenty-three subjects had both chronic mucosal disease and nasal septum deviation, while four subjects with combined allergic rhinitis and rhino-sinusitis. Although the ratio of nasal diseases was high, the prevalences of chronic mucosal disease (*p* = 0.55) and nasal septum deviation (*p* = 0.48) between HH and non-HH groups were similar.

**Table 1:** Demographic characteristics

Characteristics	Without HH	With HH	*p* value
	(*n* = 26)	(*n* = 26)	
Sex (M/F)	23/3	23/3	1
Age	47.08 ± 11.36	46.50 ± 12.10	0.45
BMI, kg/m^2^	30.03 ± 3.73	28.77 ± 3.44	0.36
ESS	12.31 ± 5.09	11.81 ± 4.95	0.78
AHI (times/h)	53.74 ± 22.32	56.65 ± 21.55	0.58
NC (cm)	40.35 ± 5.07	40.44 ± 3.33	0.29
Nasal disease	17	19	0.55

Data presented as mean ± SD or number

Abbreviations: *HH* heated humidifier, *BMI* body mass index, *ESS* Epworth Sleepiness Scale, *AHI* apnea–hypopnea index, *NC* neck circumstance

**Fig. 2:**
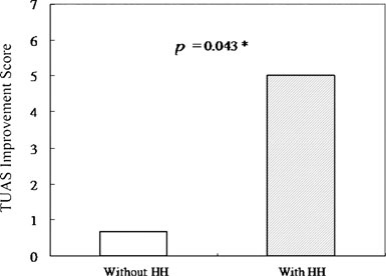
Total upper airway symptom (TUAS) improvement score is the mean reduction of naso-pharyngeal symptoms that rated on a ten-point visual analog scale. Degree of improvement in total naso-pharyngeal symptoms between subject with and without heated humidifier (*p* = 0.043). *HH* heated humidifier

Leak values, including median leak, 95^th^ percentile leak, and maximum leak, were similar in the two groups (Table 2). Furthermore, AHI reduction amounts and optimal CPAP pressure were not different between the two groups. In terms of subjective sensation, willingness to further use CPAP and feelings of sleep improvement were likewise similar (Table 2). The TUAS scores were improved in the HH group (*p* = 0.043) (Fig. 2). But individual naso-pharyngeal symptoms were not statistically different between the two groups.

**Table 2:** Objective and subjective parameters between patients with and without heated humidifiers

Parameters	Without HH	With HH	*p* value
Objective (*n* = 52)			
Optimal CPAP pressure (cm H_2_O)	9.88 ± 2.16	9.56 ± 2.26	0.66
AHI under CPAP	6.11 ± 5.08	8.65 ± 8.37	0.28
Median leak (L/s)	0.056 ± 0.036	0.059 ± 0.069	0.28
95% Leak (L/s)	0.132 ± 0.119	0.185 ± 0.183	0.17
Maximum leak (L/s)	0.445 ± 0.255	0.461 ± 0.304	0.77
Subjective (*n* = 52)			
Sleep improvement, *n* (better/similar/worse)	10/13/3	10/11/5	0.74
Willingness of use CPAP	5 ± 2.84	4.88 ± 2.58	0.385

Sleep improvement presented as subject number; other data presented as mean ± SD or number

Abbreviations: *HH* heated humidifier, *AHI* apnea–hypopnea index, *CPAP* continuous positive airway pressure

## Discussion

The present study reveals that HH during CPAP titration does not alter objective parameters such as leak, optimal CPAP pressure, and AHI reduction. In subjective sensation, HH use does not change the willingness to further use CPAP or improves sleep sensation, but it improves naso-pharyngeal symptoms. Duong’s and Wiest’s studies investigated the effect of HH on CPAP titration, and both studies prove that HH does not change objective data (i.e., optimal CPAP level and AHI reduction) [12, 23]. Furthermore, their studies also do not show that HH improves the acceptance of CPAP use. Wiest’s study did not evaluate the naso-pharyngeal symptoms of its subjects [23], while Doung’s study showed that HH does not improve naso-pharyngeal symptoms [12]. The objective data of the current study is similar to those of both studies.

There are no differences in optimal pressure level and AHI reduction between the HH and non-HH groups. However, the naso-pharyngeal symptoms are improved in the HH group, and the effects are predominantly in the mouth and nasal dryness symptoms. The difference may be due to the different patient selection. Doung’s study excludes subjects with nasal diseases and significant nasal symptoms whereas the present study has subjects with high prevalence of nasal diseases and naso-pharyngeal symptoms (nearly 70%). Therefore, the different patient populations may be the cause of the differing results.

The study by Massie et al. is the first to proposed that HH improves both compliance and naso-pharyngeal symptoms [7]. Rakotonnanahary et al. also posited the same results and revealed that patients with chronic nasal mucosal diseases, post-uvulopalatopharyngoplasty, old age, and mouth dryness due to medication achieve greater improvement [8]. Although several studies do not show the benefit of HH for CPAP compliance, most studies reveal that HH can improve naso-pharyngeal symptoms and reduce some side effects of CPAP [13–15, 24]. The reasons for the inconsistencies are not known. The diversity of studies involves different study designs and selection biases of patients. Nonetheless, more and more studies reveal that routine use of humidifiers does not alter CPAP compliance. Compliance is influenced by multiple factors, and the relationship between naso-pharyngeal symptoms and compliance is not straightforward. Thus, HH may improve naso-pharyngeal symptoms but does not help improve compliance.

Richard et al. demonstrated the presence of mouth leaks during CPAP use, which leads to a rise in nasal resistance that HH can largely prevent [25]. The authors speculate that mouth breathing generates unidirectional nasal flow that causes dryness of the nasal mucosa. It induces mucosal congestion that increases nasal resistance, which in turn also increases the possibility of mouth breathing and generates a vicious cycle. Theoretically, HH can prevent increased nasal resistance and reduce mouth breathing that can further decrease leak. Data from this study showed that HH does not reduce leak. The reason is not clear, and there is no study that evaluates the efficacy of HH on leak. Fischer’s study demonstrated the humidity level decreased when nasal mask leak developed, but compared with Richard's study the humidity level did not significantly decrease [26]. The possible explanation is that excessive mouth breathing creates a high unidirectional flow on purpose, as shown in Richard’s study performed in experimental conditions. However, most patients who receive CPAP therapy do not generate as much flow as Richard’s study and do not increase nasal resistance as much. Therefore, the present study does not demonstrate that HH reduces leak.

The limitation of this study is the optimal humidity level. Optimal humidity level is different for each subject because it is complex and relates to multiple factors, including temperature, air humidity, subjective sensation, and generated flow and pressure. The humidifier used was set to levels II to III as recommended by the manufacturer and based on clinical experience. The temperate of the sleep laboratory was controlled at 22–26°C. Although this setting might not generate optimal humidity level for all subjects, most did not present significant water condensation, and there were some vapors in the mask. There is a new model of humidifier that can automatically control humidity level, and some have been associated with heated tube to prevent water condensation. However, this type of new humidifier was not available during the study period. Further investigation is needed to evaluate whether auto-control humidifiers have better efficacy than conventional humidifiers. Another limitation is relatively small sample size might result in the low statistical power.

In conclusion, the regular use of HH, regardless of significant naso-pharyngeal symptoms, does not provide any benefits of AHI reduction, optimal CPAP level, and further CPAP acceptance. While HH improves naso-pharyngeal symptoms, its use during CPAP titration have several disadvantages, including water condensation, high noise due to water dropping in the expiration pore, cleaning of humidifier devices, and changing water for each patient. Therefore, regular use of HH during CPAP titration is not recommended.
